# A protocol of comprehensive assessment of exposure to greenspace in school children

**DOI:** 10.1016/j.mex.2021.101596

**Published:** 2021-12-03

**Authors:** Hamideh Ebrahimi Aval, Masoumeh Hashemian, Mohammad Miri

**Affiliations:** a Department of Health Education and Promotion, School of Health, Sabzevar University of Medical Sciences, Sabzevar, Iran; b Student Committee Research Center, Sabzevar University of Medical Sciences, Sabzevar, Iran; c Non-Communicable Diseases Research Center, Department of Environmental Health, School of Health, Sabzevar University of Medical Sciences, Sabzevar, Iran

**Keywords:** Strengths, Difficulties, NDVI, Park, Tree, Kids, Children, School

## Abstract

Evidence on a comprehensive greenspace exposure assessment on primary school children is scarce yet. Therefore, we aimed to assess a comprehensive greenspace exposure on primary school children and their behavioral function. We assessed different aspects of exposure to greenspace as well as behavioral function in 704 primary school children in Sabzevar, Iran, during the COVID-19 pandemic (i.e., 22 September 2020 to 10 March 2021). The greenspace indicators were including Normalized Difference Vegetation Index (NDVI) in 100, 300 and 500m buffers around children's homes based on Landsat 8 images with 30 × 30 m resolution, residential proximity to green space based on the Euclidean distance of the geocoded residential address to (i) the nearest green space of any area and (ii) the nearest green space with an area of at least 5000m^2^ (i.e., major green space) based on land use map of the study area, time spent in public and private green spaces, number of plant pots at home and visual access to greenspace based on a prepared questionnaire. The behavioral development of primary school children was assessed using a Persian online validated version of the Strengths and Difficulties Questionnaire (SDQ) filled by parents.

## Graphical abstract

**Figure:**
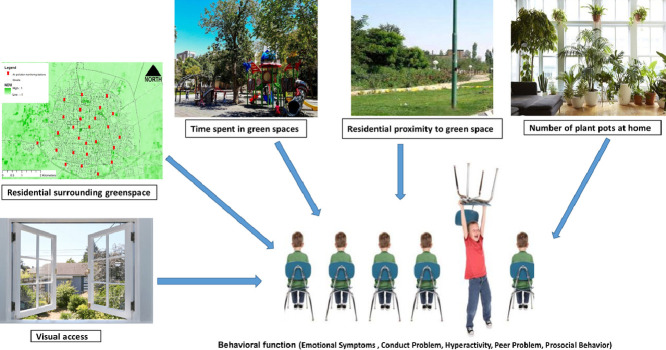

Specifications Table

**Table:** 

Subject area:	Veterinary Science and Veterinary Medicine
More specific subject area:	*Health education and Promotion, Environmental Health, Environmental Epidemiology*
Protocol name:	*Comprehensive greenspace exposure* of school children
Reagents/tools:	*ArcGIS software (version 10.5, ESRI), Strengths and Difficulties Questionnaire (SDQ), Designed questionnaire by authors*
Experimental design:	*We measured residential surrounding greenspace using stellate-based images (Landsat 8) in 100, 300 and 500m buffers around the homes and residential proximity to green space using land-use map of the study area for 704 primary school children of Sabzevar, Iran.*
Trial registration:	*N/A*
Ethics:	*This study was approved by the Ethics committee of Sabzevar University of Medical Sciences, Sabzevar, Iran (IR.MEDSAB.REC.1399.110).*
Value of the Protocol:	• *Comprehensive exposure to greenspace was investigated in primary school children.* • *Children's behavioral function was assessed using Strengths & Difficulties Questionnaires.* • *A large sample size was used to assess exposure to greenspace during the COVID-19 pandemic.*

## Description of protocol

Urbanization led to an increase in exposure to environmental pollution [1,2] and a decrease in exposure to greenspace. The available evidence reported a positive association between exposure to greenspace and behavioral development [3,4]. However, a comprehensive assessment of exposure to greenspace of primary school children in low and middle-income countries (LMICs) was not investigated yet.

### Study area

This study was conducted in Sabzevar, a city with about 240,000 population and 30 km^2^ area in the west of Khorasan province, Iran (coordinates: 36 ° 12ʹ N 57 ° 35ʹ, altitude: 977 m). According to the last census in 2016 [5], about 21,000 of the population are between 5 and 9 years old. Sabzevar is a city with four distinct seasons and has an arid climate. The annual participation in Sabzevar is about 176 mm which most of which occurred in the winter. The annual mean, minimum and maximum temperature in this city is 18 ˚C, -2 ˚C and 45 ˚C, respectively [6], [7], [8]. Sabzevar has about 55 primary schools and it should be noted that the schools of Iran are stratified by sex, i.e., about half of them are used for boys or girls, and some schools are used for both gender groups in two working shifts per day. Based on the land use map of Sabzevar, about 7.1 km^2^ have green space land use (about 27%), resulting in a 29.4 m^2^ of green space per capita [9]. The land use map of Sabzevar is shown in Fig. S1 of Supplemental Materials.

### Study setting and population

**Fig. 1:**
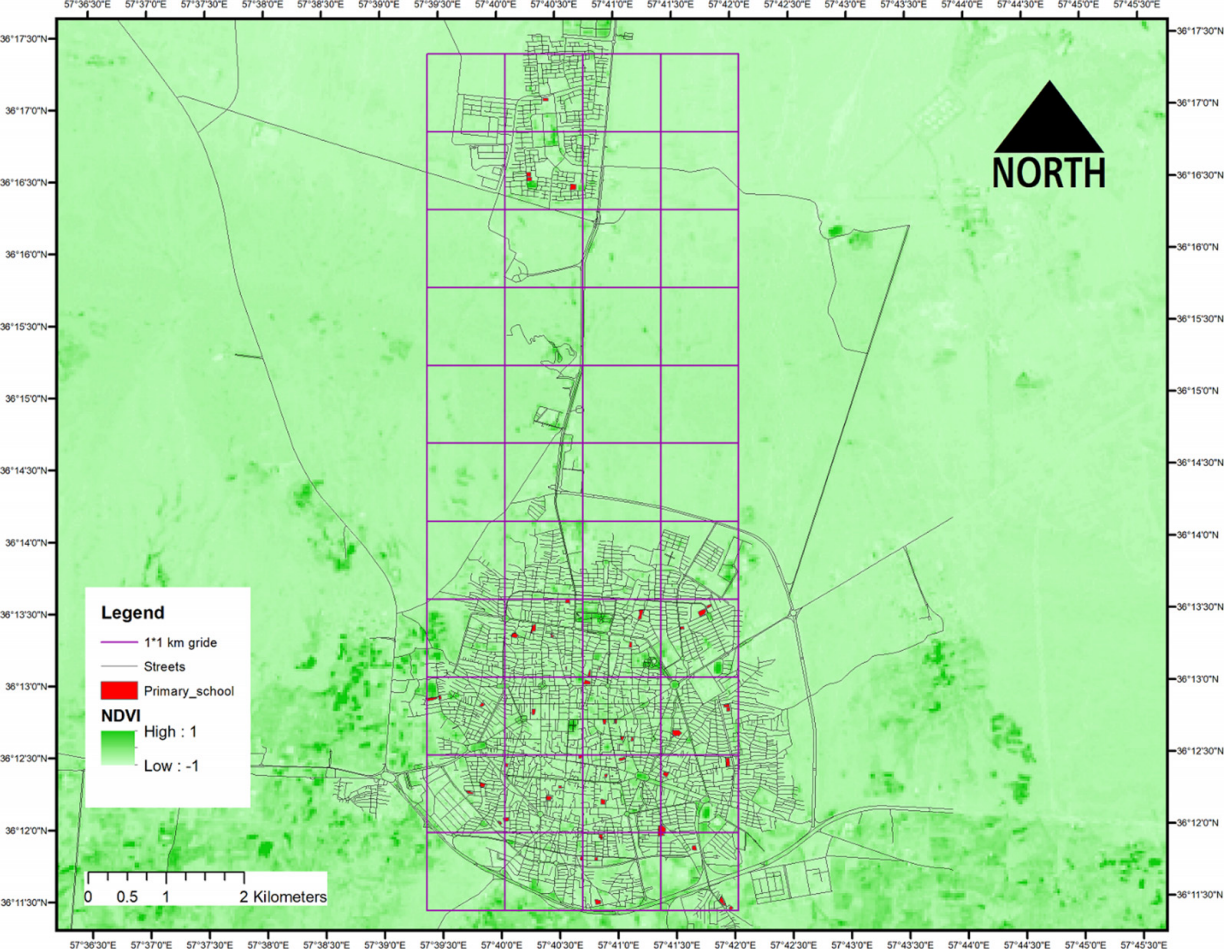
Location of the study area, streets, NDVI and 1 km grids.

**Fig. 2:**
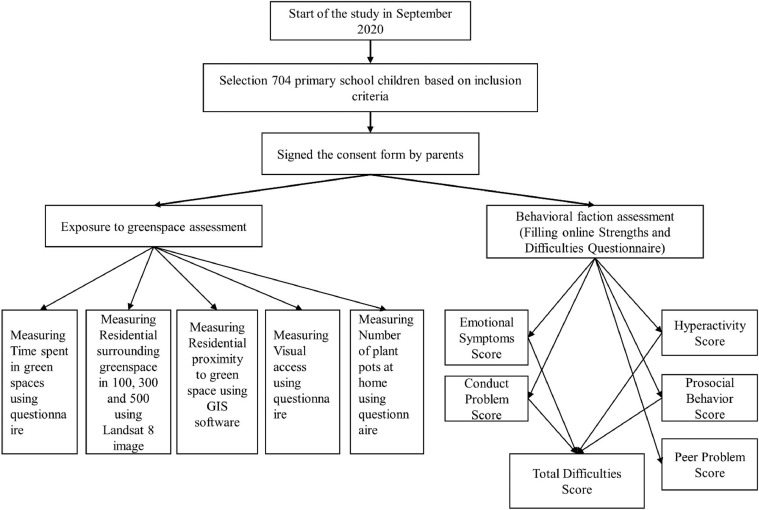
Flow diagram of the study protocol.

This cross-sectional study was based on 704 primary-school children of Sabzevar, Iran. The sampling was conducted between 22 September 2020 and 10 March 2021 from children aged 6 to 9 years old. The study area was divided into 1 × 1 km grids, and maximum two schools (one school of girls and one school of boys) were selected randomly from each grid. After that, the research steps and aims were fully described to the school officials and parents and about 60 children from each school were randomly invited to participate in our study. We included children who had no mental and physical disability, had not a diagnosed mental/sociological disease (based on children's school health records and questions from parents) and lived in Sabzevar during the study. Finally, 704 participants from 34 primary schools (20 children in average from each school) met our inclusion criteria enrolled in our study. The study was approved by the Ethics Committee of Sabzevar University of Medical Sciences, Sabzevar, Iran (IR.MEDSAB.REC.1399.110). Parents signed the consent form before beginning the study. The study area, primary schools, Normalized Difference Vegetation Index (NDVI), and grids are shown in Fig 1. Moreover, the flow diagram of the study protocol is provided in Fig 2.

### Green space exposures

#### Residential surrounding greenspace

Surrounding greenspace around children's residential home in 100, 300 500 m buffers were calculated based on a stellate image of Landsat 8 (30m × 30m resolution), downloaded on Jun 2021 [10]. The image of Landsat 8 was downloaded from U.S. Geological Survey website (https://earthexplorer.usgs.gov/). We used the Normalized Difference Vegetation Index (NDVI), a numerical indicator of greenspace, which was previously validated as a measure of characterizing green spaces. The following formula is used to measure NDVI:

(1) NDVI = (NIR - VIS)/(NIR + VIS)

where NIR and VIR are respectively land surface reflectance of near-infrared and red (visible) parts of the spectrum. It ranges from -1 to +1, where 0 indicates no vegetation and +1 means the highest possible density of green leaves. Due to the COVID-19 pandemic and absence of students from school (online education) during the study, we only calculated NDVI for their residential addresses and the NDVI of their schools was ignored [11]. We calculate the mean value of NDVI in each buffer around residential address using relevant packages (e.g., raster package) in R software.

### Residential proximity to green space

For each participant, residential proximity to green space (i.e., urban parks, street greenery, and gardens) was calculated based on the Euclidean distance of the geocoded residential address to (i) the nearest green space of any area and (ii) the nearest green space with an area of at least 5000 m^2^ (i.e. major green space). To calculate these variables, we used the land use map of Sabzevar (2016) provided by the municipality of Sabzevar and ArcGIS software version 10.5 (Environmental Systems Research Institute, Inc.) [12].

### Time spent in green spaces

Questionnaires were used to collect data on the amount of time spent in green spaces by children. For each participant, we obtained data for the time (hours/week) spent in (i) public green spaces (i.e., urban parks and public gardens) and (ii) private green spaces (i.e., private gardens, courtyards, and patios) (Table S2 of Supplemental Materials).

### Number of plant pots at home

To measure indoor exposure to greenspace, we used a question from our questionnaires asking participants to report the number of indoor plant pots at their homes (Table S2 of Supplemental Materials).

### Visual access

Visual access to greenspace was obtained using three questions: (i) Can you see plants, trees, grass, flowers, etc. from any window of your home? with possible answers being yes/no; (ii) How often do you see the vegetation through the window (s)? with possible answers being rarely, sometimes/ always; and (iii) If yes, what proportion of the window surface is covered by vegetation (If there are multiple windows, please describe the window most used)? with answers being less than 50% and ≥ 50% (Table S2 of Supplemental Materials).

## Outcome measurements

In this study, we used the Strengths and Difficulties Questionnaire (SDQ) to assess behavioral developments of primary school children. SDQ is designed in the UK. This questionnaire has several different models according to the age and the people who report the questionnaire. The questionnaire used in this study was for children from 4 to 17 years old and was completed by their parents. The five main subgroups, including hyperactivity, conduct problems, emotional symptoms, peer problems, and prosocial behavior, evaluate the behavioral functions, and the sum of the first four subgroups shows the total difficulties score [13,14]. The score for each subgroup is between 0 and 10 and for SDQ Total Difficulties Score is between 0 and 40. The Interpretations of SDQ scores are based on Table 1*.*

**Table 1:** The interpreting SDQ scores [13].

The SDQ filled by parents	This score is close to average - clinically significant problems in this area are unlikely'	'This score is slightly raised, which may reflect clinically significant problems'	'This score is high - there is a substantial risk of clinically significant problems in this area'
Total Difficulties Score	0–13	14–16	17–40
Emotional Symptoms Score	0–3	4	5–10
Conduct Problem Score	0–2	3	4–10
Hyperactivity Score	0–5	6	7–10
Peer Problem Score	0–2	3	4–10
Prosocial Behavior Score	6–10	5	0–4

In our study, the SDQ was filled by parents using an online Persian version validated by Tehranidoust et al. [15].

We double-checked the data of greenspace exposures and SDQ after identifying missing values, errors, outliers, and true (extreme or normal) values. We recalculated the greenspace indicators if we observed any missed or errors in the data of greenspace indicators. Moreover, we recalled to parents that the greenspace indicators data obtained by questionnaire or the data of SDQ for their children was unreasonable. As suggested by previous studies, we removed the outliers from our further analyses [16].

## Results

The descriptive statistics of greenspace indicators and SDQ assessed for primary school children of Sabzevar are presented in Tables 2 and 3.

**Table 2:** descriptive statistic of greenspace indicators and SDQ (continuous variables)

Variable	Mean	Standard deviation	Minimum	Maximum
NDVI in 100 buffer	0.070	0.016	0.03	0.195
NDVI in 300 buffer	0.074	0.013	0.042	0.157
NDVI in 500 buffer	0.077	0.012	0.049	0.146
Residential proximity to any greenspace (m)	230	154	0	824
Residential proximity to greenspace > 5000 m^2^ (m)	365	221	0	1062
Time spent in public green spaces (h/week)	2	3	0	24
Time spent in private green spaces (min /day)	22	54	0	420
Number of plant pots at home (n)	8	9	0	60
Total score of SDQ	11	6	0	31

**Table 3:** frequency (percent) of categorical indicators of greenspace exposure.

Variable	N	Percent (%)
Having a window with greenspace view (Yes)	386	54.8
Frequently looking at greenspace (Sometimes/always)	101	14.4
50% or more of window covered by greenspace (Yes)	158	22.44

## Declaration of Competing Interest

The authors declare that they have no known competing financial interests or personal relationships that could have appeared to influence the work reported in this paper.

## Appendix Supplementary materials

Image, application 1
