# Supplementary material for: A protocol of comprehensive assessment of exposure to greenspace in school children
Source: MethodsX. 2021 Dec 3;8:101596. doi: 10.1016/j.mex.2021.101596 (PMC8720888; doi:10.1016/j.mex.2021.101596)
Supplement: Supplementary file 1 [file mmc1.docx]

**Supplementary Materials**

**A protocol of comprehensive assessment of exposure to greenspace in school children**

**Figure S1 Page 2**

**Table S1 Page 3**


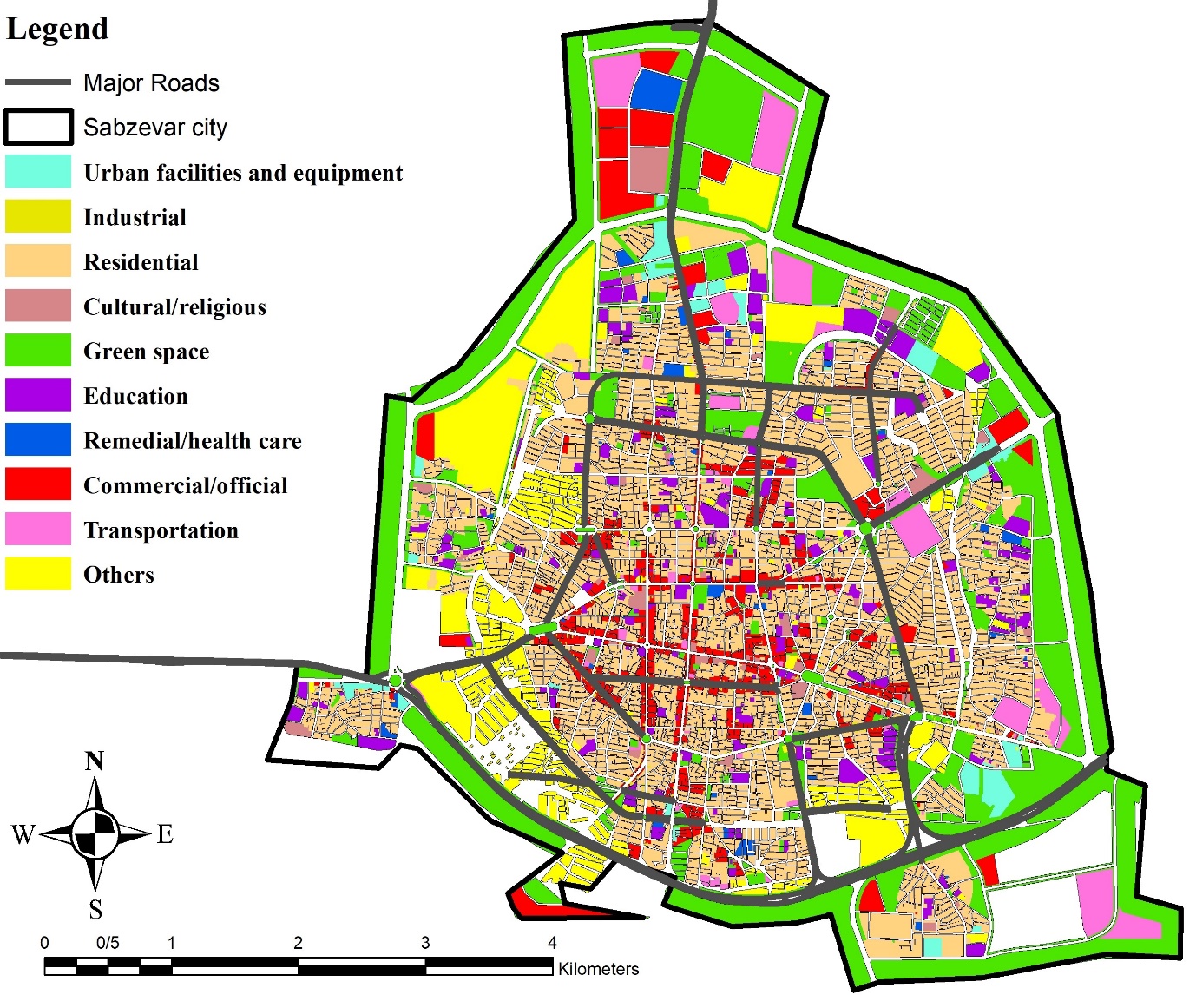


**Figure S1:** Ten different land use variables for Sabzevar

**Table S1. Greenspace questionnaire used for primary school children of Sabzevar**

| N | question | response |
| --- | --- | --- |
| 1 | How much time have you spent each week in public green space over the past year? (h/week) |  |
| 2 | For the past year, how much time have you spent each day in the green space inside your home (garden, orchard, patio, etc)? |  |
| 3 | Can you see plants, trees, grass, flowers, etc. from any window of your home? | yes/no |
| 4 | If yes, How often do you see the vegetation through the window | rarely, sometimes/ always |
| 5 | If yes (Q 3), what proportion of the window surface is covered by vegetation (If there are multiple windows, please describe the window most used)? | < 50% /  ≥ 50%. |
| 6 | How many natural pots are in your home? |  |
